# The active role of nanocarbons in electrocatalysis: recent advances in CO_2_ conversion

**DOI:** 10.3389/fchem.2025.1745268

**Published:** 2025-12-18

**Authors:** Daniele Giusi, Veronica Costantino, Viviana Amoroso, Claudio Ampelli

**Affiliations:** Department of Chemical, Biological, Pharmaceutical and Environmental Sciences (ChiBioFarAm), University of Messina, ERIC aisbl and CASPE/INSTM, Messina, Italy

**Keywords:** carbon-metal interfaces, CO_2_ electroreduction, gas-diffusion electrodes, nanocarbon materials, triple-phase boundary

## Abstract

The electrocatalytic reduction of CO_2_ (CO_2_RR) powered by renewable energy offers a promising strategy to mitigate climate change while generating valuable fuels and chemicals. Achieving high performance in this process strongly depends on the properties of the electrode materials and the overall electrode architecture. In this context, nanocarbon materials, generally used as supports, are far from being inert; they can actively influence CO_2_RR by stabilising adsorbed intermediates and directing reaction pathways through their hydrophobicity, porosity and defective structure. Unlike most reviews that focus exclusively on the active metal phase, this mini-review highlights the emerging dual role of nanocarbons (acting both as substrates and as active components) in determining catalytic activity and selectivity. It summarises recent advances in CO_2_RR using nanocarbon-based materials, including both metal-free and hybrid systems, and discusses how doping and interfacial engineering enhance CO_2_ activation, product selectivity and process efficiency. Gas-diffusion electrodes incorporating nanocarbon architectures improve mass transport and triple-phase boundary formation (gas-solid-liquid interface), enabling high current densities and multi-carbon product generation. These aspects demonstrate that tuning nanocarbon properties is essential for developing efficient and scalable CO_2_RR electrodes, thereby advancing sustainable carbon utilisation technologies.

## 1 Introduction

Expensive metal-based catalysts have long dominated catalytic processes. However, the widespread use of noble-metal-based materials in electrochemical reactions, while effective, is hindered by high costs, restricting their scalability (Liu et al., 2022; Tian et al., 2022; Zhang et al., 2024). In contrast, nanocarbon materials, such as graphene and carbon nanotubes (CNTs), have emerged as viable and environmentally friendly alternatives to costly metal-based catalysts for electrochemical conversion, owing to their excellent electrical conductivity, high surface area and outstanding thermal stability (Askins et al., 2021; Faraji et al., 2023; Liu et al., 2019). Although pristine carbon is generally chemically inert, its catalytic performance can be strongly enhanced by introducing new active sites through dopant and defect engineering, while harnessing the tunability offered by different carbon allotropes (Bhardwaj et al., 2023; Zheng X. et al., 2023).

In addition to their intrinsic activity, nanocarbon materials can be assembled into hybrid architectures, exploiting synergistic interactions with molecular species and metal centres. Metal-carbon hybrid materials, including molecular complexes immobilised on nanocarbon supports and single-atom catalysts (SACs), exhibit remarkable performance in key electrochemical reactions by enhancing charge transfer, stabilising intermediates, and reducing the amount of precious metals required (Ji et al., 2025; Qi et al., 2023; Qu et al., 2023).

In parallel, several efforts have been devoted to the development of carbon-based metal-free electrocatalysts (C-MFECs) (Hu and Dai, 2016; Hu et al., 2021; Liu et al., 2019; Zhai et al., 2023; Zhao et al., 2019b). These earth-abundant, low-cost and chemically stable materials show remarkable catalytic activity when doped or functionalised with heteroatoms such as O, N, and P, with wide applications in electro-, photo-, and thermo-catalytic reactions, including the hydrogen evolution reaction (HER), oxygen reduction reaction (ORR), oxygen evolution reaction (OER), CO_2_ reduction reaction (CO_2_RR) and nitrogen reduction reaction (NRR) (Centi and Ampelli, 2024; Hu and Dai, 2016; Hu et al., 2021; Li et al., 2020; Zhao et al., 2019a). C-MFECs combine high conductivity, large surface area and porosity, and structural versatility (from 0D to 3D). They can be synthesised by scalable methods such as chemical vapour deposition, chemical modification and ball milling (Shi L. et al., 2024; Zhai et al., 2024), and can even be derived from biomass (Polidoro et al., 2023). The introduction of heteroatoms modulates the catalytic properties by altering the electronic structure of the carbon skeleton, allowing fine-tuning of structure-activity relationships at the atomic level.

**FIGURE 1:**
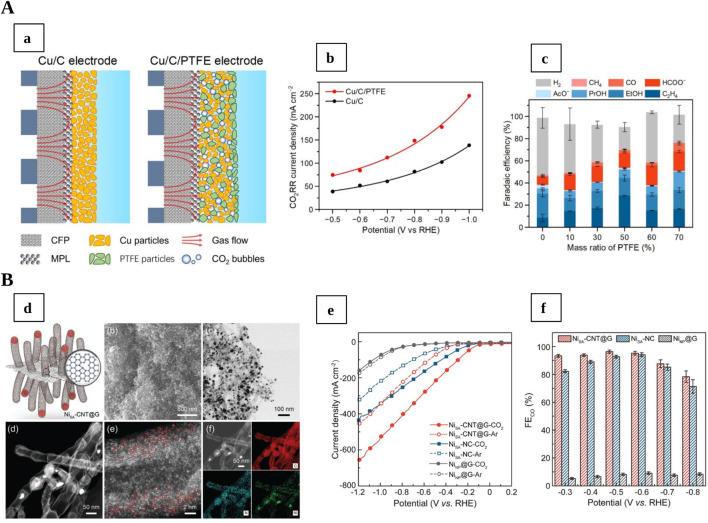
**(A)** (a) Schematic representation of non-modified GDE (left) and GDE with PTFE (right) to create a hydrophobic environment; (b) CO_2_RR current densities for Cu/Cu (blue line) and Cu/C/PTFE modified catalyst (red line) at different applied potentials; (c) Faradaic efficiencies at different PTFE mass ratio (%), Reproduced with permission of (Shi K. et al., 2024). **(B)** (d) morphological characterisation of Ni-CNT@G catalysts; (e) Current densities and (f) CO Faradaic efficiencies for Ni-CNT@G compared to reference samples, Reproduced with permission of (Jiang et al., 2024).

In the context of CO_2_ electroreduction, optimised nanocarbon materials, either metal-free or hybrid systems, enable the formation of carbon monoxide (CO), formate, methane, and multi-carbon products, through active sites such as pyrrolic and pyridinic N atoms, defect-adjacent carbons and oxygenated groups (Shang et al., 2022; Zhu et al., 2022). Beyond their intrinsic catalytic properties, nanocarbon-based gas-diffusion electrodes (GDEs) have been widely used to overcome CO_2_ mass-transport limitations and increase the local CO_2_ concentration at the catalyst interface, resulting in higher Faradaic efficiencies (Ampelli et al., 2023; Ronsisvalle et al., 2025). While surface functionalization and multi-doping strategies are essential for improving reaction kinetics and promoting synergistic effects, the performance of GDEs (Figure 1A) can be controlled by tuning the hydrophobicity and porosity of the electrode, thereby optimising the triple-phase interface (gas reactant–solid electrocatalyst–liquid electrolyte) and mitigating flooding (Rabiee et al., 2024; Shi K. et al., 2024; Wu et al., 2024).

In this mini-review, we summarise the most up-to-date advances in CO_2_RR using nanocarbon-based materials, including both metal-free and hybrid systems. Unlike most reviews that focus exclusively on the metal phase or on general aspects of carbon materials independent of CO_2_ activation, this work emphasises the emerging multifunctional role of nanocarbons in determining catalytic activity and selectivity. We provide an explicit and comparative discussion of nanocarbons as: (i) intrinsically active, metal-free catalytic sites, (ii) microenvironment regulators in GDE architectures controlling mass transport and wettability, and (iii) electronically and chemically coupled supports in metal-carbon hybrid systems. We discuss the design principles of nanocarbon-based electrocatalytic materials and electrode architectures, and how doping and interfacial engineering enhance CO_2_ activation, product selectivity and process efficiency. These aspects are essential for developing efficient and scalable CO_2_RR electrodes, thereby advancing sustainable carbon utilisation technologies. This review does not cover non-electrochemical CO_2_ conversion routes, photocatalytic-only systems, nor general nanocarbon literature related to oxygen reduction reaction (ORR)/oxygen evolution reaction (OER) or thermal catalysis, thereby providing a focused and clearly defined scope.

## 2 Nanocarbon-based electrode architectures and gas-diffusion electrodes

The design of gas-diffusion electrodes (GDEs), most based on nanocarbon-based substrates, is crucial for achieving high activity and selectivity in CO_2_RR, as it governs the delicate balance between gas, liquid, and solid phases at the catalytic interface. GDEs are engineered to overcome CO_2_ mass-transport limitations and to establish efficient triple-phase boundaries where CO_2_, electrolyte, and active sites coexist (Giusi et al., 2025). However, the long-term operation of these systems is often hindered by issues such as flooding, electrolyte penetration and degradation of hydrophobicity, which reduce CO_2_ accessibility and favour competing HER (Lai et al., 2023). Optimising the physical and chemical properties of GDEs, particularly their surface wettability, pore accessibility and hierarchical porosity, is therefore essential to sustain CO_2_ supply, mitigate flooding and maintain high electrocatalytic performance.

Recent studies have explored innovative GDE architectures based on nanocarbon supports to address these limitations. For instance, Chen et al. (2025) developed hollow-fiber GDEs (HFGDEs) incorporating hydrophobic PTFE within Zn-nanosheet-coated Cu hollow nanofibers, achieving a ∼39% increase in CO partial current density and a 66.7% reduction in surface water coverage compared to unmodified electrodes. The hydrophobized HFGDEs improved CO_2_ distribution and stabilised the triple-phase boundary, suppressing the HER. To enhance durability against hydrophilization and electrolyte flooding, (Jiang et al., 2024), designed a CNT/graphene-based GDE embedding Ni-N_x_ single-atom sites (Figure 1B). Its hierarchical and superhydrophobic porous structure facilitated efficient mass transport and stabilisation of the triple-phase boundary, yielding a Faradaic Efficiency (FE) for CO of 96.3% at a current density of 406.85 mA cm^−2^. Similarly, Wang M. et al. (2023), reported hydrophobized PTFE-supported Ni nanofiber GDEs with nearly 100% FE for CO and >273 h stability in membrane electrode assembly (MEA). This carbon-based architecture boosted mechanical stability and electrical conductivity, improving CO_2_ supply and active-site utilisation, and underscoring the critical role of triple-phase boundary stability.

Beyond CO production, tailored GDE architectures can promote C–C coupling and multicarbon product formation. Tandava et al. (2025) achieved an FE to ethylene (C_2_H_4_) ≥ 70% and an overall C_2+_ FE above 90% at −0.55 V vs. RHE, with a current density of 250 mA cm^−2^, by sputtering Cu onto porous PTFE membranes containing oxide/carbon additives. The carbon-based PTFE/Cu architecture enhanced the CO_2_ local concentration, stabilising the triple-phase interface and promoting C_2_H_4_ at ultra-low potentials. A different approach by Yamaguchi et al. (2024) introduced an Al conductive interlayer on a hydrophobic polymer support, enabling the use of resistive Cu_2_O catalysts while maintaining >50% FE for C_2_H_4_. Likewise, Lu et al. (2025), demonstrated that optimising the thickness of a defective carbon layer in CuSn(OH)_6_@C (21 nm) enhanced C–C coupling and ethanol formation, reaching 65.8% FE for ethanol at 300 mA cm^−2^. Under acid conditions, where carbonate formation is minimised but CO_2_ diffusion becomes limiting (Sun et al., 2024), fabricated a GDE containing a superhydrophobic ultrathin microporous Cu layer, enhancing CO_2_ diffusion and stabilising the triple-phase boundary, achieving 87% FE for C_2+_ products at a partial current density of 1.6 A cm^−2^.

Flooding mitigation and hydrophobic grading represent critical strategies to sustain high current densities. Recently, da Silva et al. (2024), reported a Cu Mesh-PTFE sandwich electrode that reached 500 mA cm^−2^ while ensuring efficient CO_2_ delivery and maintaining a stable triple-phase boundary, showing enhanced C_2_H_4_ formation by tuning mesh porosity. Li et al. (2023) integrated Ni-single-atom/Cu catalysts into hydrophobic-graded GDEs, minimising electrolyte flooding, enhancing ^*^CO coverage and C_2_H_4_ selectivity while suppressing carbonate precipitation in MEA electrolysers. Similarly, Xiong et al. (2023), developed a CoPc–CNT–ODA composite GDE with atomically dispersed Co sites and robust hydrophobicity, achieving 97.7% FE for CO at a partial current density of 154.8 mA cm^−2^ with stable operation over 12 h. In this system, the carbon-architecture controlled water availability and preserved the stability of the triple-phase interface. Finally, Min et al. (2023), reported a heteroatom-free porous carbon membrane enriched in intrinsic defects (HDPCM) that enhanced CO_2_ adsorption and activation. After hydrophobic treatment, this material achieved 81.1% FE for CO at −0.66 V and 50 mA cm^−2^. Beyond structural optimisation (Sun et al., 2025), examined the spatial distribution of CO_2_RR active regions in Bi/Ag-based electrodes, revealing that the catalytic zone extends 150–600 nm from the PTFE substrate and is influenced by humidity, current density and hydrophobicity, key parameters for next-generation GDE design.

## 3 Carbon-based metal-free catalysts

Carbon-based metal-free electrocatalysts (C-MFECs) have emerged as promising candidates for preparing electrodes for CO_2_RR, combining sustainability, abundance and structural tunability. Unlike metal catalysts, these systems rely on intrinsic defects, heteroatom doping and tailored electronic structures to activate CO_2_ and control selectivity. By adjusting dopant type, porosity and local coordination environment, it is possible to modulate adsorption energies and stabilise key intermediates such as *COOH, thereby promoting efficient and selective CO_2_RR. This section discusses recent advances in heteroatom-doped carbons, porous frameworks, and multi-doped or redox-active systems, emphasising the structure-function correlations that govern their activity and stability.

### 3.1 Heteroatom doping strategies

Heteroatom doping is one of the most effective routes to induce activity in otherwise inert carbon lattices by modifying charge density, spin distribution and local bonding configurations. Among these, nitrogen doping has proven particularly versatile in generating active sites and addressing selectivity. Yang et al. (2025) developed N-doped porous carbon nanosheets (NPCNs) derived from coal tar pitch, showing that pore size and pyridinic-N content significantly affect CO_2_RR performance, achieving a maximum FE for CO of 92% at −0.7 V vs. RHE with a partial current density to CO of 3.5 mA cm^−2^. Similarly, Narimatsu et al. (2025), combined zeolite templating with recarbonization to tune N-species distribution, reaching 76% FE for CO and confirming by DFT that the synergistic effect of N-sites and adjacent C-atoms stabilises the *COOH intermediate. Han et al. (2025) further demonstrated that coupling N-doping with mesoporous architecture enhances CO_2_RR kinetics and suppresses HER, achieving 95% FE for CO at −0.50 V vs. RHE due to improved local pH and *COOH stabilisation.

**FIGURE 2:**
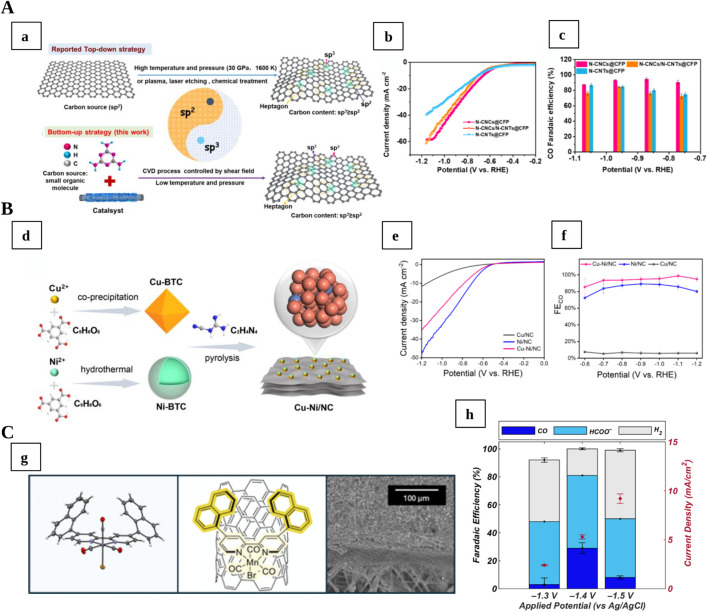
**(A)** (a) Representation of nitrogen-doped carbon nanomaterial with high amount of high sp^3^/sp^2^ defects, Reproduced with permission of (Tan et al., 2024); (b) current density and (c) CO Faradaic efficiencies at different potentials for N-doped carbon nanochains with abundant defects (N-CNCs-CFP), compared to reference samples. **(B)** (d) scheme preparation for Cu−Ni/ NC synthesis; (e) Current densities and (f) CO Faradaic efficiencies of Cu−Ni/NC compared with references, Reproduced with permission of (Cao et al., 2024). **(C)** (g) Crystal structure and SEM image of a cambered bipyridyl ligand with extended aryl immobilised on MWCNTs; (h) Faradaic efficiencies and current density for Mn(nap-bpy)MWCNT-1 sample, Reproduced with permission of (Lee et al., 2025).

The formation of specific N hybridisation is also critical in addressing the catalytic behaviour. Tan et al. (2024) prepared necklace-like nitrogen-doped carbon nanochains (N-CNCs) supported on carbon nanofibers, rich in sp^3^ defects (31.2%) and nitrogen (23.6%), achieving 95.9% FE for CO at −0.86 V vs. RHE with a current density of 23.3 mA cm^−2^ (Figure 2A). Xiao et al. (2024) introduced piperazine into a high-density aminal-linked COFs enriched in sp^3^-hybridised nitrogen, reaching up to 19.1% FE to C_2_H_4_, among the highest reported for COF-based systems. Jiao et al. (2025) identified pyridinic-N as the primary active site using hierarchical carbon nanocages (hCNCs), observing 83.6% FE at 9.5% N, while S-doping promoted HER, describing the dopant-activity correlation. An et al. (2024) explored the role of amine-N sites embedded in the carbon lattice, preparing ultrathin amine-functionalized carbon nanosheets and achieving 98% FE for CO at 55 mA cm^−2^ with 50-h stability in flow-cell operation. Fu et al. (2023) analysed N-doped biochar catalysts with tunable properties, showing that defect density, porosity, graphitisation degree and surface hydrophobicity are key factors in determining activity, even more important than the N-content or surface area. Their optimised materials reached 94.9% FE for CO.

Apart from nitrogen, oxygen- and phosphorous- doping strategies have been explored to modulate charge distribution and intermediate adsorption energies. Wang et al. (2025) used a covalent carbon nitride template to synthesise O-C_2_N catalysts enriched with C-O-C ether moieties, achieving 94.8% CO selectivity over 20 h Zhao et al. (2025) reported a 3D printing P/N co-doped carbon with a tunable H_2_:CO ratio (0.58–3.65) and a maximum FE for CO of 63.5% at −0.7 V vs. RHE, with N and P atoms acting as electron acceptor-donor pairs. Similarly Zheng W. et al. (2023), fabricated a P/N-doped 3D mesh-like metal-free aerogel with uniformly distributed active sites, yielding 85% FE for CO at 100 mA cm^−2^. ATR-FTIR confirmed the synergistic role of pyrrolic-N active centres and P atoms, favouring H_2_O dissociation and *COOH formation.

### 3.2 Porous carbon frameworks

Beyond chemical doping, the architectural control of porosity and defect density provides another route to enhance CO_2_ adsorption, diffusion, and active-site exposure. Ramírez-Valencia et al. (2025) synthesised carbon xerogel microspheres containing tunable amounts of eco-graphene, enabling adjustable syngas ratios and achieving 89.2% FE for CO. Narzary et al. (2023) introduced porous polyimides (pPIs) with CO_2_ capture capacities up to 14 wt%, reaching 91% and 85% FE for formate and methanol, respectively. A ternary-doped porous carbon catalyst (SePNC) developed by Feng et al. (2024) delivered 94.8% FE for CO, retaining over 95% of the initial FE and 80% of the current density during 12 h electrolysis. The combination of hierarchical porosity and high surface area (970 m^2^ g^−1^) improved both mass transport and exposure of catalytically active sites.

### 3.3 Multi-doping and redox systems

Introducing multiple heteroatoms can further enhance CO_2_RR by coupling different electronic effects and the presence of defects. Sun et al. (2023) reported Se, P, B and N tetra co-doped nanocarbons with a BET area of 1023.6 m^2^ g^−1^ and rich porosity, achieving 96.2% FE for CO at −0.5 V vs. RHE, maintaining 82.7% of the current density after 10 h of continuous electrolysis. In a different approach (Barman et al., 2024), synthesised metal-free redox-active triphenylamine (TPA)-derived covalent organic nanosheets, reaching 51.6% FE for methanol at only 210 mV overpotential, attributed to the high density of accessible redox sites exposed on 2D nanosheets. Still Fu et al. (2024), designed hydrophobic metal-free carbon quantum dots (CQDs) enriched in basic Lewis sites, which promoted *COOH formation and water activation, producing CH_4_ with 52% FE at 178 mA cm^−2^. Both these studies demonstrate that combining multi-doping, defect engineering, and redox-active structures represents a powerful strategy to achieve selective, durable, and scalable CO_2_RR electrodes using entirely metal-free carbon frameworks.

### 3.4 Structure-activity relationships in doped nanocarbons

Heteroatom-doped nanocarbon materials exhibit enhanced performance due to modulated electronic properties, charge density and binding energy for CO_2_RR intermediates, thereby driving the activity and selectivity. N-doping, mainly in pyridinic form, typically favours CO selectivity, creating electron-rich sites that stabilise the ^*^COOH. O-doping can promote ^*^HCOO^−^ (formate) formation *via* synergistic interaction of oxygenated functional groups (Yang et al., 2019). On the contrary, P-doping and multi-doping can favour CO or multi-carbon products, suppressing the HER by tuning the electronic structure, especially when combined with N in co-doped architectures (He et al., 2023; Shahid et al., 2025). These insights stress the importance of rational dopant engineering in the development of nanocarbon-based catalysts.

Representative metal-free based catalysts discussed throughout Section 3, including their structure, main products and performance, are summarised in Table 1.

**TABLE 1:** Representative carbon-based metal-free electrocatalysts (C-MFECs) for CO_2_RR.

Carbon type/Architecture	Dopants/Active sites	Main products	Electrolyte	Cell configuration	Faradaic efficiency (FE)	Voltage vs. RHE	Current density (total or partial)	Role of heteroatoms	References
​					%	V	mA cm^−2^		
N-doped porous carbon nanosheets (NPCNs)	N (Pyridinic-N enriched)	CO	0.1 M KHCO_3_	n.d.	92	−0.7	J_CO_ = −3.5	Activity boosted by pyridinic-N + mesopore-enhanced mass transport	Yang et al. (2025)
Necklace-like N-doped carbon nanochains (N-CNCs) on CFP	N (23.6 at%), high sp^3^ defects (31.2%)	CO	0.1 M KHCO_3_	H-type cell	95.9	−0.86	J_tot_ = −23.3	High sp^3^/sp^2^ ratio + N-doping enhance CO selectivity	Tan et al. (2024)
3D crosslinked N,P-doped carbon aerogel (P@NCA)	N (pyrrolic-N as active site) P (synergistic)	CO	1.0 M KHCO_3_	Flow cell	85	−1	J_tot_ = −100	N–P synergy enhances *COOH formation and *CO desorption	Zheng et al. (2023a)
Oxygen-substituted C2N (O-C2N)	O-ether (on C2N framework, intrinsic N)	CO	0.1 M KHCO_3_	Flow cell	94.8	−0.6	J_CO_ = −2.75	CO selectivity governed by exocyclic O-substituted N–C–O–C moieties	Wang et al. (2025)
Porous polyimide (pPI) porous organic framework	n.d.	HCOO^−^, CH_3_OH	0.1 M KHCO_3_	H-type cell	91 (formate), 85 (methanol)	0.03 (formate), −0.26 (methanol)	J_tot_ < −0.050	BXJ pore-engineering tunes CO_2_ capture and product selectivity	Narzary et al. (2023)
Se, B, P, N multi-doped carbon (Se-BP-N-C)	Se, B, P, N (multi-doping)	CO	0.5 M KHCO_3_	Flow cell	96.2	−0.5	J_tot_ ∼ −2 mA mg^−1^	Multi-doping enhances selectivity; high stability over 10 h electrolysis	Sun et al. (2023)
Hydrophobic carbon quantum dots (CQDs)	High Lewis basic sites	CH_4_	1 M KOH	Flow cell	52	−2.4	J_tot_ = −178	Lewis basic sites enhance CO_2_ adsorption, *COOH → *CO conversion, proton availability from H_2_O	Fu et al. (2024)

## 4 Hybrid carbon-metal systems

As described in the previous sections, carbon-based materials offer unique advantages as active electrocatalysts for CO_2_RR due to their high electrical conductivity, chemical robustness, and tunable surface and electronic properties. Their defect-rich structure, heteroatoms and curved lattices provide abundant sites for reactant adsorption and activation, while also serving as versatile scaffolds for the incorporation of metallic species. In hybrid carbon-metal systems, the carbon phase assumes a dual role, acting both as a catalytically active matrix and as a conductive, chemically interactive support that modulates the behaviour of metal centres. At the nanoscale, strategies such as controlled doping, alloying, strain engineering or single-atom dispersion enable a fine modulation of the local electronic environment, facilitating efficient charge transfer and stabilising key reaction intermediates. This intimate interplay between carbon and metal species results in synergistic effects, maximising metal utilisation and unlocking multiple catalytic pathways.

### 4.1 Metal-carbon interfaces and single-atom catalysts (SACs)

The performance of hybrid catalysts is often dictated by the structure and chemistry of the metal-carbon interface, where charge redistribution and orbital hybridisation modulate reaction energetics. Wang X. et al. (2023) reported a formate-selective Cu-Bi carbon composite (Bi-Cu/HMCS), employing a “copper-bridge” strategy to promote *p-d-p* hybridisation at the C-Cu-Bi junction. This interfacial configuration stabilised HCOO^*^ intermediate, achieving 100% FE for formate at 100 mA cm^−2^ and 53.8% energy efficiency in a solid-state device. Yu et al. (2024) developed a Cu-btca (btca = benzotriazole-5-carboxylic acid) MOF active nanostructure under acidic conditions, obtaining 51.2% FE for C_2_H_4_ and 81.9% for multi-carbon products due to efficient C-C coupling at Cu sites. A Cu-Ni alloy supported on N-doped nanocarbon, synthesised by Cao et al. (2024), achieved nearly 99% FE for CO, where the Cu-Ni synergy and hydrophobic support efficiently suppressed HER and balanced COOH^*^ and CO^*^ desorption (Figure 2B).

Beyond alloy systems, single-atom catalysts (SACs) supported on nanocarbons offer atomic-level control of coordination environments and maximise metal efficiency. Wen et al. (2024) developed a Ni_1_–N–C material *via* microwave-assisted hydrolysis of Ni–ZIF-8, achieving 96% FE for CO with a partial current density of 1.06 A cm^−2^, attributed to its mesoporous, defect-rich carbon framework. Using electrospinning (Wang et al., 2024), fabricated highly active Fe-N-C catalysts, reaching 97% FE for CO at −0.51 V vs. RHE and a current density of 5.3 mA cm^−2^, with DFT calculation confirming COOH^*^ stabilisation at Fe–N_4_ sites. Jiao et al. (2024) anchored Au single atoms on N-doped carbon-nanocages (hNCNC) for syngas production (H_2_/CO = 0.4–2.2), achieving a remarkable mass activity of 3391 A g^−1^_(Au)_ at −1 V vs. RHE, outperforming the behaviour of Au nanoparticles on the same support, due to different responses of CO_2_RR and HER on single-atom Au sites. Likewise Liu et al. (2025), reported hierarchical ZIF-8 Ni SACs with 82% FE for CO and a partial current density of 492 mA cm^−2^ at −3.4 V, and 120 h stability. DFT calculations revealed the role of N atoms in the second coordination layer to facilitate ^*^COOH formation. Choi et al. (2025) demonstrated dynamic Cu single-atom evolution on nanocarbon supports (Cu-SAC-NC), forming nearly 100% metallic Cu nanograins during operation. The conductive carbon backbone promoted C-C coupling, leading to a fivefold increase in FE for C_2_^+^.

Together, these studies highlight how precise interfacial design, from alloying to single-atom dispersion, addresses CO_2_RR selectivity and stability by coupling electronic tuning, defect engineering and carbon support properties.

### 4.2 Ligand functionalization

Ligand functionalization offers an additional level of molecular precision for tuning activity and selectivity in hybrid metal-carbon systems. Functional ligands modulate the local electronic structure, coordination geometry and metal-support interactions, often promoting more efficient charge transfer and stabilising key intermediates.

Guo et al. (2025) covalently anchored cobalt phthalocyanine (CoPc) onto carbon nanotubes *via* ball milling, leading to a 40% increase in methanol FE compared to non-covalent analogues, due to enhanced CoPc dispersion, charge-transfer dynamics and π–π coupling. Lee et al. (2025) immobilised Mn(bpy)(CO)_3_Br complexes on multiwalled carbon nanotubes (MWCNTs), obtaining >92% FE for CO at 16.5 mA cm^−2^. The high activity arose from π–π interactions and a ligand “cambering effect” that optimised orbital alignment (Figure 2C). Li et al. (2024) prepared phthalocyanine-based molecular catalysts, supported on carbon support (CoPc@NC), showing that electron-withdrawing substituents such as–CN groups enhanced catalytic performance by modulating the substrate-catalyst charge redistribution. Similarly Wei et al. (2025), designed a pyrene-modified Co-quaterpyridine complex anchored on carboxyl-functionalized MWCNTs, achieving nearly 100% FE for CO and a high turnover number. The π–π interaction between the pyrene and CNT backbone facilitated charge delocalisation, while the axial Co-O coordination tuned the adsorption strength of intermediates.

In summary, hybrid carbon-metal systems represent an emerging class of advanced CO_2_RR electrocatalysts, combining the electrical conductivity, structural versatility and chemical tunability of nanocarbon frameworks with the specific catalytic activity of metal active centres. Through precise control of interfacial chemistry, atomic dispersion and ligand coordination, these materials can influence reaction pathways towards value-added carbon products with enhanced selectivity, durability and energy efficiency, thus delineating a viable route towards scalable electrocatalytic CO_2_ valorisation.

### 4.3 General role of metal-carbon interfaces

In general, in hybrid carbon-metal catalysts, the carbon framework acts both as a conductive matrix and as active support, shaping the metal centres’ reactivity. Second-sphere heteroatom doping, M-N coordination, lattice defects, local strain and carbon structure (e.g., porosity and curvature) are key factors modulating the local charge distribution, influencing metal cluster dispersion and mobility, ultimately governing intermediate binding energies (Song et al., 2024). In the SAC systems, a precise carbon-metal engineering maximises the metal utilisation, enabling multiple catalytic pathways, especially when incorporated in multi-component systems in which the carbon matrix favours a synergistic interaction between SACs and metal clusters, thus promoting tandem catalytic pathways (Wu et al., 2023). The synergy of nanocarbon matrices and metal sites highlights the importance of the nanocarbons in controlling the local environment, governing the metal-carbon charge transfer, driving CO_2_RR selectivity. Table 2 provides a selection of representative hybrid-carbon metal systems presented in Section 4, highlighting carbon support, metal type and general performance.

**TABLE 2:** Representative hybrid carbon–metal systems.

Metal/SAC	Carbon support	Main products	Electrolyte	Cell configuration	Faradaic efficiency (FE)	Voltage vs. RHE/Cell voltage	Current density (total or partial)	Interfacial design	References
​					%	V	mA cm^−2^		
Bi–Cu	Hollow mesoporous carbon spheres	Formate	n.d.	Solid-state electrolyte device	91.3	Cell voltage = 2.5	J_Tot_ = −143	Cu-bridge for p–d–p modulation	Wang et al. (2023b)
Cu−Ni	N-doped nanocarbon (hydrophobic, MOF-derived).	CO	0.5 M KHCO_3_	H-type cell	98.8	−1.1 V	J_CO_ = −27.6	Synergistic Cu–Ni sites; hydrophobicity suppresses HER; optimized COOH* formation and CO* desorption	Cao et al. (2024)
Ni single-atom (Ni-N-C, ZIF-8 modified)	Nitrogen-doped carbon nanofibers (NCNF) with hierarchical porosity	CO	0.5 M K_2_SO_4_ (pH = 1.0)	MEA electrolyzer	82	Cell voltage = 3.38	J_CO_ = −492	ZIF-8 creates hierarchical porosity for mass transfer; N second-shell doping promotes *COOH formation	Liu et al. (2025)
Cobalt phthalocyanine (CoPc, molecular catalyst)	Carbon nanotubes (CNT, covalently anchored, ball-milled)	CH_3_OH	0.5 M KHCO_3_	H-cell based	15.1	−1.3	J_CH3OH_ = −9.9	CoPc covalent anchoring, improved charge transfer	Guo et al. (2025)
Cobalt quaterpyridine complex (Coqpy, molecular catalyst)	MWCNT (COOH; π–π anchoring)	CO	0.5 M KHCO_3_	Customized single electrolytic cell	97.7	−0.63	n.d.	Axial Co–COOH coordination + pyrene π–π coupling	Wei et al. (2025)
Cu-btca MOF	Porous MOF network formed *in situ*; phase transformation during electrolysis	C_2_H4, C_2+_	3.0 M KCl and 0.05 M H_2_SO_4_ (pH 1)	Flow cell	51.2 (C_2_H_4_) 81.9 (C_2+_)	−2.5	J_Tot_ = −300	Two adjacent Cu sites facilitate C–C coupling	Yu et al. (2024)

## 5 Summary and outlook

This mini-review has examined the active and multifaceted role of nanocarbon-based materials in CO_2_RR. Nanocarbons exhibit a dual functionality, as both catalytically active components and conductive substrates, where their structure, composition and interfacial properties strongly influence activity and selectivity. Through heteroatom doping, multi-doping strategies, and defect engineering, the carbon framework can modulate the local electronic environment and stabilise key intermediates, thus enhancing CO_2_ activation, suppressing the hydrogen evolution reaction, and favouring the formation of multi-carbon products.

In gas-diffusion electrode (GDE) configurations, the design of nanocarbon frameworks with controlled porosity, wettability, and triple-phase boundary is fundamental to improving CO_2_ transport and availability at the catalytic interface. Hierarchically structured and hydrophobic nanocarbons, whether as metal-free or integrated in hybrid architectures, showed efficient CO_2_ conversion at industrially relevant current densities (>200 mA cm^−2^).

For carbon-based metal-free electrocatalysts (C-MFECs), defect engineering and heteroatom incorporation, particularly with nitrogen and oxygen functionalities, have emerged as key strategies to modulate the electronic density and local charge distribution, thus enhancing CO_2_ adsorption and activation. Despite these advances, identifying the precise nature and dynamic evolution of the active sites under electrochemical operation remains a major challenge, limiting predictive catalyst design.

In hybrid carbon–metal systems, the incorporation of metal species (ranging from nanoparticles to single atoms) into conductive nanocarbon matrices induces strong interfacial synergistic effects. These interactions regulate charge transfer, active-site geometry, and intermediate stabilisation. Ligand functionalisation and interfacial engineering further expand the tunability of these systems, allowing fine control over reaction pathways. Among the various approaches, single-atom catalysts anchored on nanocarbon substrates represent one of the most promising architectures, offering atom-efficient utilisation and high selectivity toward C_2_+ products, although their operational durability and resistance to metal aggregation remain critical issues for practical implementation.

Future research should focus on establishing quantitative correlations between dopant configuration, defect structure, and catalytic activity, supported by *in situ* and operando characterisation techniques integrated with advanced theoretical modelling. In parallel, progress in electrode design, particularly in engineering robust triple-phase boundaries, will be crucial to bridge the gap between laboratory-scale performance and industrial requirements.

Beyond flooding and hydrophilization, long-term GDE performance is hindered by carbonate and salt precipitation within the porous scaffold, becoming critical at high current densities and leading to mechanical instability and destabilization of the triple-phase boundary. Furthermore, structural breakdown and carbon corrosion can occur at extreme potentials, especially when operating in acidic media where oxidation dissolution is more pronounced, while alkaline media introduce issues related to carbonate formation, affecting long-term CO_2_RR performance.

Sustainability and techno-economic considerations, supported by TEA/LCA analysis, remain essential in assessing whether complex nanocarbon architectures, ranging from COFs to simple biomass-derived carbons, are economically viable pathways for large-scale CO_2_RR implementation.

By consolidating the most recent advances in the field, this review aims to provide guidance for the development of next-generation nanocarbon-based electrodes capable of achieving selective, efficient and scalable CO_2_ electroreduction under realistic operating conditions.
